# Towards Evidence-Based Weaning: a Mechanism-Based Pharmacometric Model to Characterize Iatrogenic Withdrawal Syndrome in Critically Ill Children

**DOI:** 10.1208/s12248-021-00586-w

**Published:** 2021-05-17

**Authors:** Sebastiaan C. Goulooze, Erwin Ista, Monique van Dijk, Dick Tibboel, Elke H. J. Krekels, Catherijne A. J. Knibbe

**Affiliations:** 1 Division of Systems Biomedicine and Pharmacology, Leiden Academic Centre for Drug Research, Leiden University, Einsteinweg 55, 2333 CC Leiden, The Netherlands; 2 LAP&P Consultants BV, Leiden, The Netherlands; 3 Pediatric Surgery, Erasmus Medical Center-Sophia Children’s Hospital, Rotterdam, The Netherlands; 4 Division of Nursing Science, Department of Internal Medicine, Erasmus Medical Center, Rotterdam, The Netherlands; 5 Department of Clinical Pharmacy, St. Antonius Hospital, Nieuwegein, The Netherlands

**Keywords:** Pharmacometrics, Pediatric, Iatrogenic withdrawal, Morphine, Fentanyl

## Abstract

For the management of iatrogenic withdrawal syndrome (IWS) in children, a quantitative understanding of the dynamics of IWS of commonly used opioids and sedatives is lacking. Here, we introduce a new mechanism-based pharmacokinetic-pharmacodynamic (PKPD) modeling approach for studying IWS in pediatric clinical datasets. One thousand seven hundred eighty-two NRS_withdrawal_ scores of IWS severity were analyzed, which were collected from 81 children (age range: 1 month–18 years) that received opioids or sedatives by continuous infusion for 5 days or more. These data were successfully fitted with a PKPD model consisting of a plasma and a dependence compartment that well characterized the dynamics of IWS from morphine, fentanyl, and ketamine. The results suggest that (1) instead of decreasing the infusion rate by a set percentage at set intervals, it would be better to lengthen the weaning period when higher infusion rates are administered prior to weaning; (2) for fentanyl specifically, the risk of IWS might be lower when weaning with smaller dose reductions every 12 h instead of weaning with greater dose reductions every 48 h. The developed PKPD model can be used to evaluate the risk of IWS over time and the extent to which it is affected by different weaning strategies. The results yield hypotheses that could guide future clinical research on optimal weaning strategies. The mechanism-based PKPD modeling approach can be applied in other datasets to characterize the IWS dynamics of other drugs used in pediatric intensive care.

**Graphical abstract:**

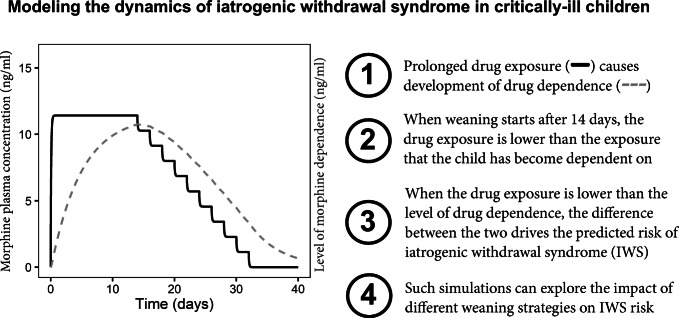

**Supplementary Information:**

The online version contains supplementary material available at 10.1208/s12248-021-00586-w.

## INTRODUCTION

Opioids and sedatives are crucial in ensuring critically ill children’s well-being and comfort (1). Even though prolonged treatment may be required for clinical reasons, drug dependency and iatrogenic withdrawal syndrome (IWS) may occur during weaning of these drugs and subsequently hamper patient recovery (2, 3). Effective weaning strategies are crucial to prevent IWS itself but may also reduce situations where concerns about IWS lead to the undertreatment of pain and distress in critically ill children (1, 4).

The reported incidence of IWS in different studies varies widely (5 to 87%) (3). Prolonged treatment and high cumulative drug doses are the most commonly reported risk factors for IWS (1, 3). While risk factors can identify patients who need weaning, they do not inform us how weaning should be performed. Thus, different institutions apply a large variety of weaning strategies (5, 6). To reduce the risk of IWS in clinical practice, we need weaning strategies based on a patient’s characteristics and type of drug and dosing history over time.

Population pharmacokinetic-pharmacodynamic (PKPD) modeling approaches could help to develop weaning strategies for IWS. In general, PKPD models use dosing information to predict drug concentrations over time and then relate these concentrations to the drug’s effect (7). In this case, the effect would be an adverse effect—the occurrence of IWS. Rather than treating the risk of IWS as a static percentage, a PKPD modeling approach treats it as a dynamic risk that can change over time and is affected by the treatment before and during weaning. More specifically, these models can be used to simulate the risk of IWS over time in different scenarios, based on an individual’s medication history—consisting of different opioids and sedatives in different doses and durations—and other patient-level risk factors. On the basis of this risk of IWS over time, different weaning strategies can be compared, and the most appropriate strategy can be selected.

In the underlying mechanism of IWS, drug treatment plays a dual role. Prolonged drug treatment may cause dependence, which puts the patient at risk of IWS (8). In a drug-dependent patient, ongoing treatment with the drug will prevent the occurrence of IWS, while stopping drug treatment too fast will likely incite IWS (1, 6). There are currently no PKPD models that can characterize the development of dependence over time during prolonged drug treatment, as well as the risk of IWS and disappearance of dependence over time during the weaning phase.

In this work, we propose a novel, mechanism-based PKPD model that is able to describe how exposure to selected opioids and sedatives over time contributes to the development and disappearance of drug dependence and the risk of IWS over time. The model was developed using data from a previous study in which the Sophia Observation withdrawal Symptoms scale (SOS) was validated in a PICU population of children who received continuous infusions of opioids or sedatives for 5 days or more (9). We performed model simulations to generate new hypotheses for optimal weaning strategies.

## METHODS

### Clinical Study

We performed a secondary analysis on data from a previous prospective observational study by Ista *et al*. (9) that included children from term neonates up to children of 18 years of age who received opioids or sedatives by continuous infusion for 5 days or more. The study was approved by the local institutional review board, which waived the need for parental informed consent. The study considered children admitted to the ICU between March 2009 and September 2010 in the Erasmus MC-Sophia Children’s Hospital. From the 154 children included in the original study, we excluded all infants younger than 1 month and those receiving extracorporeal membrane oxygenation treatment, due to a lack of literature information on the population pharmacokinetics of the investigated opioids and sedatives in these populations.

The measure of IWS severity used in the current study is the NRS_withdrawal_ score, a numeric rating scale used by a member of the nursing staff to rate the IWS severity, ranging from 0 (no IWS) to 10 (worst IWS possible). This rating considers both the observed withdrawal-associated symptoms, as well as contextual factors such as the (duration of) use of opioids and sedatives, and the possibility of co-occurring pain, under-sedation or delirium (9). NRS_withdrawal_ scores above 3 are considered to reflect IWS that requires pharmacological intervention. A total of 1782 NRS_withdrawal_ scores were available for the development of the mechanism-based PKPD model for IWS.

### Mechanism-Based PKPD Model for Iatrogenic Withdrawal

We first calculated, on the basis of the administered dosages to each individual subject, the plasma concentration over time profiles of the main opioids and sedatives (morphine, fentanyl, methadone, midazolam, lorazepam, clonidine, ketamine, and propofol) administered during the PICU stay. For these calculations, we used appropriate pediatric population pharmacokinetic models of these drugs that characterized the drug disposition and drug clearance in our patient population (10–20). The bioavailability and rate of absorption for extravascular routes of administration were taken from pediatric population pharmacokinetic models or, if these were not available, from non-compartmental analyses in children (14, 21–23) or analyses in adults (24–32). Supplemental Material 1 provides a detailed overview of the population PK models and relevant literature references. This document also contains the number of drug doses and the routes of administration for each of the opioids and sedatives considered.

To characterize the development and disappearance of drug dependence over time, a novel mechanism-based IWS modeling approach was developed. For each drug, the model contains a hypothetical “dependence compartment” which equilibrates with the drug’s central pharmacokinetic (plasma) compartment with an estimated rate constant *k*_dep_. This constant controls the rate at which patients become dependent on a particular drug, as well as the rate at which patients become less drug-dependent after drug treatment is reduced or discontinued:

1 $$ \frac{d{C}_{\mathrm{dep}\mathrm{endence}}}{dt}={k}_{\mathrm{dep}}\ast \left({C}_{\mathrm{plasma}}-{C}_{\mathrm{dep}\mathrm{endence}}\right) $$

where *C*_dependence_ represents the hypothetical concentration that the patient is dependent on and *C*_plasma_ is the drug concentration in plasma. The “concentration shortage,” defined as the difference between *C*_dependence_ and *C*_plasma_, was used to drive the IWS severity using a linear relationship (Eq. 2). Drugs were considered to have no effect on IWS when *C*_dependence_ ≤ *C*_plasma_. The IWS severity caused by a single drug j (Effect_j_) was defined according to Eq. 2:

2 $$ {\mathrm{Effect}}_{\mathrm{j}}=\left\{\begin{array}{c}{\mathrm{Slope}}_j\ast \left({C}_{\mathrm{dependence}}-{C}_{\mathrm{plasma}}\right),\kern0.5em if\ {C}_{\mathrm{dependence}}>{C}_{\mathrm{plasma}}\\ {}0\kern15.25em ,\kern0.5em if\ {C}_{\mathrm{dependence}}\le {C}_{\mathrm{plasma}}\end{array}\right. $$

where Effect_j_ represents the contribution to IWS by drug j, and slope_j_ is the estimated slope parameter for this particular drug. Because *C*_dependence_ and *C*_plasma_ change over time, the Effect_j_ also changes over time. Non-linear alternatives (such as the *E*_max_ function) to Eq. 2 were also tested. Overall IWS severity was modelled as additive effects of each drug included in the model (Eq. 2) to an estimated baseline:

3 $$ {\lambda}_i={Baseline}_i\cdotp {e}^{\eta_i}+{\sum}_{j=1}^{Ndrugs}{Effect}_j $$

where *λ*_*i*_ is the overall IWS severity of individual *i* at a particular time; *Baseline*_*i*_ is the estimated baseline IWS severity of a typical individual; *η*_*i*_ represents the *post hoc* estimate of how much the baseline IWS severity of individual *i* deviates from the typical baseline; and *Ndrugs* is the number of drugs included in the IWS model.

We also explored whether drugs used to manage IWS during weaning (e.g., clonidine) could lower the IWS severity caused by other drugs, whether drugs would influence the IWS caused by drugs from the same class (e.g., morphine lowering the fentanyl-associated withdrawal or vice versa), and whether the development of tolerance (in addition to dependence) could be modelled with an additional compartment. The model equations for this are given in the Supplemental Material 2.

The method proposed by Plan *et al*. was used to relate the model-predicted *λ*_*i*_ to the probability of observing a particular NRS_withdrawal_ score (33). This method uses a generalized truncated Poisson model to account for the fact that only integer values of 0–10 are possible outcomes of the NRS_withdrawal_ scale. To account for the correlation between subsequent observations within an individual, the model included a Markovian inflation of the probability of observing NRS_withdrawal_ scores that are within two points of the previously observed NRS_withdrawal_ score. We estimated the probability inflation of having the same score when the previous score was zero (π0|0), the probability inflation of having the same score for different previous scores (π0|x), and the probability inflation of having a score that is ±1 points or ±2 points away from the previous score (π±1, π±2). The final model code is provided in Supplemental Material 3.

### PKPD Model Development and Evaluation

During model development, PKPD models were fitted with NONMEM 7.3 using the Laplace conditional estimation method. For the final PKPD model, an expectation-only step of the Importance Sampling (IMP) method—using the final parameter estimates from the Laplace method—was performed to obtain a covariance matrix of the parameter estimates (34).

Model development started with a base model in which none of the administrated drugs affected IWS severity. To this base model, using stepwise model development, the effects of different drugs were added to the model according to the Eqs. 1–3, to see if doing so would improve the fit of the observed NRS_withdrawal_ data. Effects of drugs that did not improve the fit of the data were not included. Competing models were compared using the objective function value for nested models or the Akaike information criterion. The patient characteristics age, sex, Pediatric Risk of Mortality Score III (PRISM III), and diagnosis category were tested as covariates for the inter-individual variability in baseline IWS severity (Eq. 3) using a stepwise forward inclusion approach and included in the model if this resulted in a significant (*p*<0.01) reduction in the objective function value.

Two techniques were used to evaluate whether the goodness-of-fit of the model and its predictions, by comparing them to the observed NRS_withdrawal_ data. Mirror plots (35) were used to compare the predicted and observed frequencies for each possible NRS_withdrawal_ score, as well as the frequencies of specific transitions between consecutive observations. The expected NRS_withdrawal_ at a particular observation is calculated as:

4 $$ Expected\ {NRS}_{\mathrm{withdrawal}}=\sum \limits_{j=0}^{10}j\times p\left( score=j\right) $$

where *p(score=j)* is the individual *post hoc* model-predicted probability of observing an NRS_withdrawal_ score equal to *j* at a particular observation. Additionally, residuals were calculated as the difference between the observed NRS_withdrawal_ score and the expected NRS_withdrawal_ score. The residuals were then plotted against time and versus the expected NRS_withdrawal_ to visually inspect these plots for the presence of trends that might be indicative of model misspecification.

Using the PKPD model, we simulated different scenarios to confirm that the model’s behavior is in agreement with clinical knowledge on the dynamics of IWS: abrupt discontinuation of a continuous infusion increases the risk of IWS, but this risk can be reduced by slowly weaning off the continuous infusion (1, 6). To aid interpretation of the simulation results, we calculated the probability of having an NRS_withdrawal_ score above 3 over time to reflect the risk of IWS. Then, to generate new hypotheses on optimal weaning strategies in different clinical scenarios, additional simulations were performed of a typical patient’s IWS risk over time (i.e., without inclusion of inter-individual variability on baseline IWS severity).

## RESULTS

### Clinical Study Data

In the present study, we included data from 81 children (see Table I), which included 1782 NRS_withdrawal_ scores collected during a median PICU stay of 16 days (IQR 10–34), with 198 (11.1%) of the scores above 3, indicating the presence of IWS. In 42 (52%) patients, IWS was observed at one or more occasions during the study. The median number of times IWS was observed in a patient was 1 (IQR 0-3); in 13 patients (16%) IWS was observed six times or more.

**Table I:** Characteristics of the Patients (*n*=81) Included in This Analysis

Variable	Median (interquartile range) or *n* (%)	Range
Age (months)	22 (6–75)	1.4–228
Weight (kg)	11 (6–20)	2.4–70
Male sex	41 (51)	–
Diagnosis category		
Respiratory	32 (40)	–
Cardiac/circulatory	15 (19)	–
Congenital defects	1 (1)	–
Surgical/postoperative	11 (14)	–
Trauma	7 (9)	–
Other	15 (19)	–
Length of stay PICU (days)	16 (10–34)	5–96
Pediatric Risk of Mortality Score III	9 (4–14)	0–30
Cumulative drug doses per patient during the study^a^		
Morphine (mg/kg)	1.5 (0.73–2.85)	0–16.3
Fentanyl (mcg/kg)	11 (3.5–29)	0–2252
Midazolam (mg/kg)	41 (23–78)	0–385
Lorazepam (mg/kg)	0 (0–0.19)	0–27.1
Ketamine (mg/kg)	2.2 (0–101)	0–579
Propofol (mg/kg)	9.2 (2.9–44)	0–837
Clonidine (mcg/kg)	0 (0–42.8)	0–1065
Methadone (mg/kg)	0 (0–0)	0–51.4
Number of different opioids and sedatives received^b^	5 (4–6)	2–8

*PICU* pediatric intensive care unit

^*a*^Cumulative doses are given as intravenous equivalents. Extravascular doses are transformed into intravenous equivalents using the bioavailability of the population PK models used

^*b*^Out of the eight opioids and sedatives studied: morphine, fentanyl, midazolam, lorazepam, ketamine, propofol, clonidine, methadone

The patients received a wide range of cumulative doses of opioids and sedatives (Table I). Some of the studied drugs were given at least once in almost all patients (i.e., morphine (93%), fentanyl (94%), midazolam (99%), and propofol (84%)), while other drugs were given to fewer patients (i.e., ketamine (60%), clonidine (44%), lorazepam (37%), and methadone (6%)). Patients received a median of 5 (IQR 4–6) different types of opioids and analgesics, out of the total of 8 opioids and sedatives that were studied here.

### Mechanism-Based PKPD Model for Iatrogenic Withdrawal

To the NRS_withdrawal_ data of the patients, we fitted our mechanism-based PKPD model that includes for each drug a hypothetical dependence compartment that equilibrates with the central (plasma) compartment. The concentration in this hypothetical compartment represents the concentration (*C*_dependence_) that the patient is dependent on at a particular time point, and this is driven by an estimated rate constant k_dep_ and the concentration in the plasma compartment (Eq. 1), which is in its turn dependent on the drug dosages given over time. When the plasma concentration of a drug is lower than the *C*_dependence_, the IWS risk predicted by the model increases with increasing difference between *C*_dependence_ and *C*_plasma_ (Eq. 2).

**Fig. 1:**
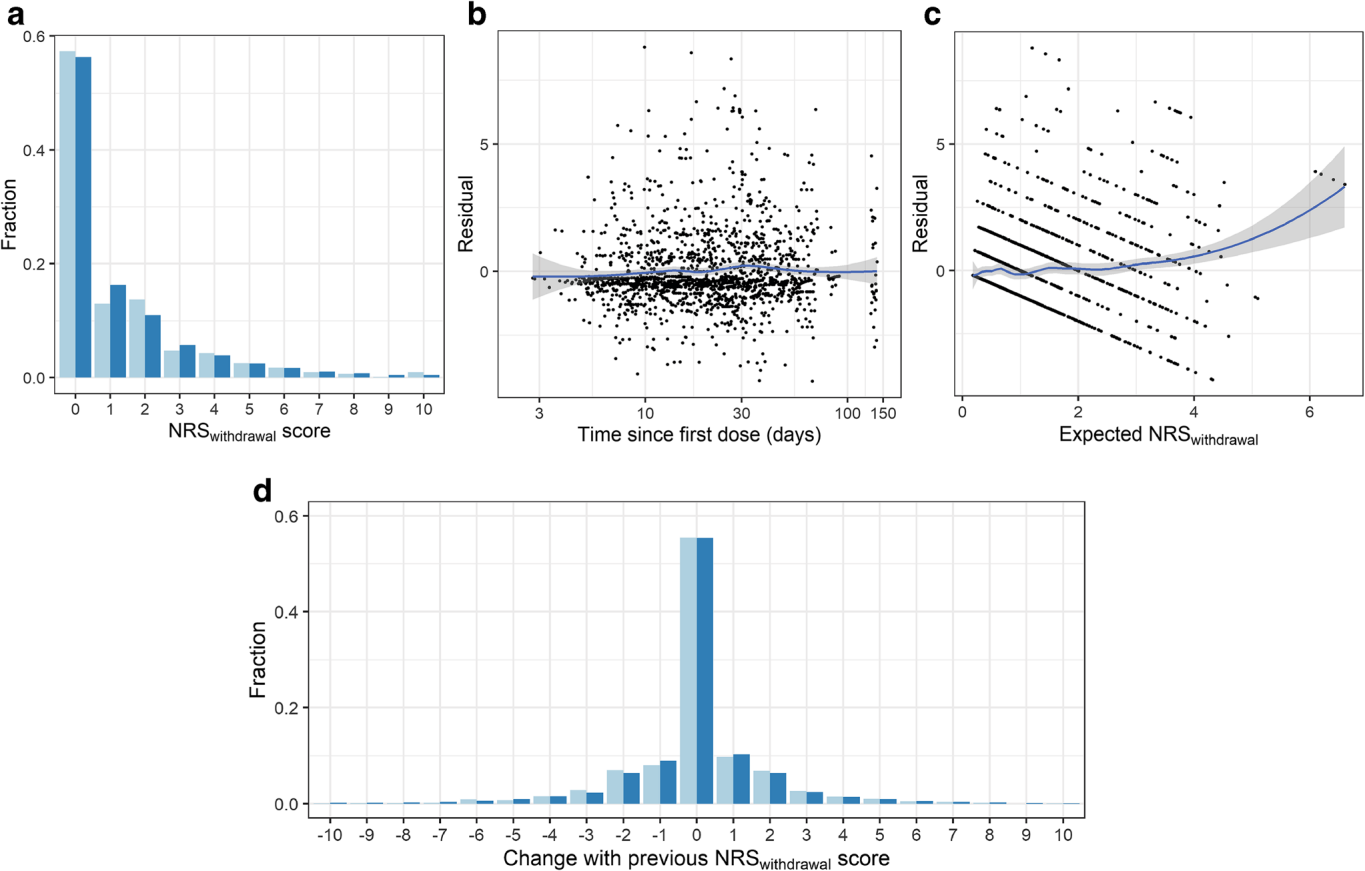
Model diagnostic plots of final PKPD model. **a** and **d** Mirror plot of observed (light blue) and predicted (dark blue) frequencies of NRS_withdrawal_ scores **a** and their transitions between consecutive observations **d**. The predicted frequencies are calculated as the mean of the individual *post hoc* estimates of the probabilities at each observation. Transition is calculated as follows: current NRS_withdrawal_ score – previous NRS_withdrawal_ score. **b** and **c** Each point represents one observation, where the residual is calculated as the difference between the observed NRS_withdrawal_ score and the expected NRS_withdrawal_ score. The black line with grey area represents a LOESS smoother of the data with its 95% confidence interval, respectively

Model development started with a base model in which none of the drugs affect IWS, and after development of the statistical error model, drugs were added to the model in a stepwise manner (Supplemental Table S1). The dynamics of IWS and dependence could be quantified for three drugs, which significantly improved the fit of the NRS_withdrawal_ data: fentanyl (*p*<0.001), morphine (*p*=0.04), and ketamine (*p*=0.006). The estimated values for *k*_dep_, the model parameter that controls the rate of dependence development and disappearance over time, varied widely between these drugs, with a high rate constant that indicates fast dependence for fentanyl (*k*_dep_ = 0.242 h^−1^) and lower rate constants for morphine and ketamine (*k*_dep_ = 0.00848 h^−1^ and 0.0180 h^−1^, respectively). Midazolam, clonidine, lorazepam, methadone, and propofol were not added to the model, as they did not significantly improve the model fit. No significant influence of patient’s age, sex, PRISM III, and diagnosis category was identified (*p*>0.01). The parameter estimates of the final mechanism-based IWS model are provided in Table II. There was good agreement between the predictions of the model and the observed NRS_withdrawal_ data over time in the mirror plots (Fig. 1 a and d) and goodness-of-fit plots (Fig. 1b). Individual fit plots of six randomly selected subjects are provided in Supplemental Material 5.

**Table II:** Parameter Estimates of Mechanism-Based Iatrogenic Withdrawal Syndrome (IWS) Model

Parameter	Estimate (95% CI^a^)
Baseline NRS_withdrawal_ (Eq. 3)	
Typical baseline Inter-individual variability baseline (CV%)	0.564 (0.384–0.828) 114% (60.6–170)
Fentanyl (Eqs. 1 and 2)	
*k*_dep_ (h^−1^) Slope_fentanyl_ (ml ng^−1^)	0.242 (0.109–0.527) 5.93 (2.73–12.87)
Morphine (Eqs. 1 and 2)	
*k*_dep_ (h^*−*1^) Slope_morphine_ (ml ng^*−*1^)	0.00848 (0.00378–0.0190) 0.0687 (0.0276–0.171)
Ketamine (Eqs. 1 and 2)	
*k*_dep_ (h^*−*1^) Slope_ketamine_ (ml mcg^*−*1^)	0.0180 (0.00668–0.0482) 1.59 (0.768–3.29)
Overdispersion and Markov probability inflation	
δ π0|0 π0|x π±1 = π±2	0.428 (0.350–0.506) 0.401 (0.321–0.486) 0.216 (0.179–0.258) 0.0698 (0.0422–0.113)^b^

*CI* confidence interval; NRS_withdrawal_, score of withdrawal severity on a numerical rating scale; CV%, coefficient of variation; δ, coefficient of dispersion parameter; π0|0, probability inflation of observing the same score as before if the previous score was zero; π0|x, probability inflation of observing the same score as before if the previous score was not zero; π±1, probability inflation of observing a score that is 1 point higher or lower than the previous score; π±2, probability inflation of observing a score that is 2 points higher or lower than the previous score

^*a*^Calculated from standard error of estimates generated from NONMEM’s covariance step

^*b*^π±1 and π±2 were constrained to be equal to each other

**Fig. 2:**
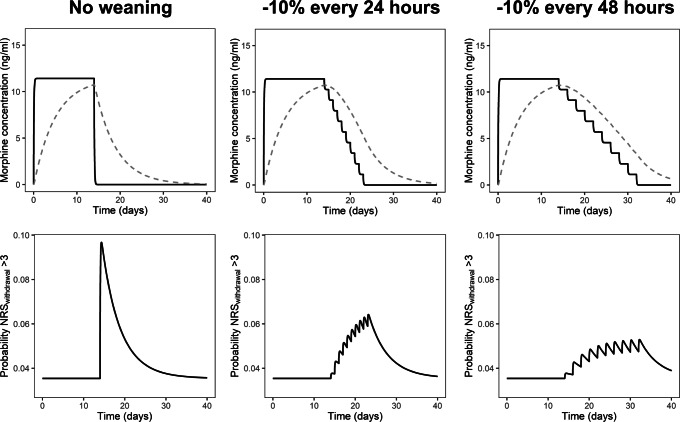
Illustration of the risk of iatrogenic withdrawal syndrome (IWS) over time predicted by the mechanism-based IWS model for different weaning strategies after 14-day treatment period with continuous intravenous morphine at 20 mcg kg^−*1*^ h^−*1*^ in a typical patient with a 10-kg body weight. The top row panels show the simulated morphine concentrations in plasma (*C*_plasma_, solid black line) and morphine concentrations that the child has become dependent on (*C*_dependence_, dashed grey line). The bottom row panels show the predicted probability of an NRS_withdrawal_ score above 3, which indicates IWS

Figure 2 illustrates that the performance of the final model is in agreement with clinical knowledge on IWS: i.e., abrupt discontinuation of a continuous infusion increases the risk of IWS, but this risk can be reduced by slowly weaning off the continuous infusion (1, 6). In each panel of Fig. 2, simulations are shown for a 10-kg patient receiving continuous intravenous morphine infusion of 20 mcg kg^−1^ h^−1^ during 14 days. The morphine concentration in plasma (solid line) quickly reaches steady state in this period, while the concentration that the child has become dependent on (*C*_dependence_, dashed line) slowly increases during the first 14 days. If the morphine infusion is abruptly stopped on day 14 (Fig. 2, left column), there is a large difference between *C*_plasma_ and *C*_dependence_, which results in an almost threefold increase in the risk of IWS (bottom row, left column) compared to baseline and this risk decreases over time as *C*_dependence_ decreases. By lowering the morphine infusion by 10% of the initial rate every 24 or 48 h, the *C*_plasma_ and *C*_dependence_ are lowered more gradually, resulting in lower risk of IWS (Fig. 2, middle and right column). Additionally, a more gradual weaning strategy (10% of initial rate every 48 h) results in a slightly lower peak risk of IWS compared to the weaning strategy of reduction with 10% of initial rate every 24 h.

### Weaning Simulations

**Fig. 3:**
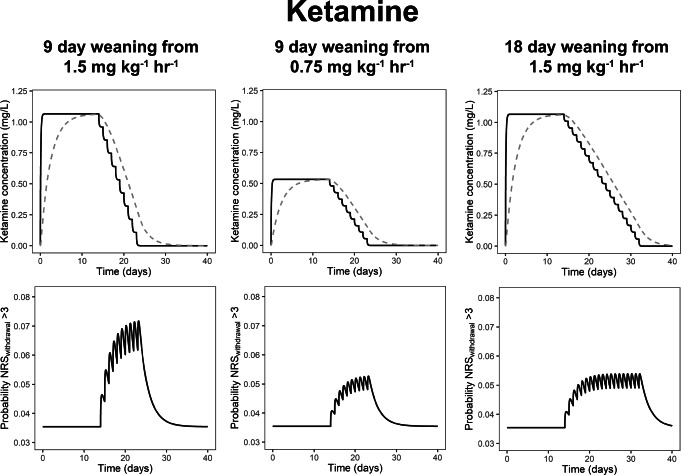
The impact of the ketamine infusion rate (1.5 or 0.75 mg kg^−*1*^ h^−*1*^) during a 14-day treatment period and weaning duration (9- or 18-day weaning) on the risk of iatrogenic withdrawal syndrome (IWS) during weaning in a typical patient with a 10-kg body weight. The top row shows the simulated ketamine concentrations in plasma (*C*_plasma_, solid black line) and ketamine concentrations that the child has become dependent on (*C*_dependence_, dashed grey line). The bottom row shows the predicted probability of an NRS_withdrawal_ score above 3, which indicates IWS. In all scenarios simulated here, the time between consecutive weaning steps is 24 h

We then performed simulations to generate hypotheses on optimal weaning in different scenarios. For simplicity sake, these scenarios only included monotherapy with intravenous administration. Figure 3 illustrates for ketamine the impact of the infusion rate during the 14-day treatment period on the risk of IWS. In all panels, a typical 10-kg child receives continuous ketamine infusion for 14 days, followed by a weaning period. If a 9-day weaning period is used, the IWS risk is higher for a 1.5 mg kg^−1^ h^−1^ infusion (left column) than for a 0.75 mg kg^−1^ h^−1^ infusion (middle column) during the treatment period. When, however, a longer 18-day weaning period (right column) is used after the 1.5 mg kg^−1^ h^−1^ infusion, the risk of IWS is similar to the scenario with a lower infusion rate but a shorter 9-day weaning period. The same phenomenon occurs in simulations with morphine and fentanyl (Supplemental Material 6 and 7, respectively)

**Fig. 4:**
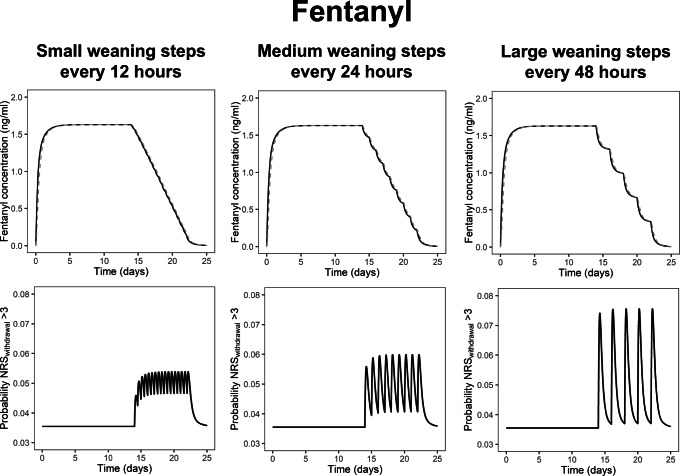
The impact of time between weaning steps and weaning step size on the risk of iatrogenic withdrawal syndrome (IWS) after 14-day treatment period with continuous intravenous fentanyl at 1.5 mcg kg^−*1*^ h^−*1*^ in a typical patient with a 10-kg body weight. In all three scenarios, the time between the first reduction in the fentanyl infusion and the complete discontinuations of the fentanyl infusion is 8 days; the scenarios only vary in time between weaning steps and the infusion rate reduction in these steps. The top row shows the simulated fentanyl concentrations in plasma (solid black line) and fentanyl concentrations that the child has become dependent on (dashed grey line). Due to the high dependence rate of fentanyl (*k*_dep_ = 0.265 h^−*1*^), *C*_plasma_ and *C*_dependence_ closely follow each other. The bottom row shows the predicted probability of an NRS_withdrawal_ score above 3, which indicates IWS

Figure 4 compares for fentanyl the impact on IWS risk of using frequent, small weaning steps versus less frequent, but greater weaning steps. In all scenarios shown in Fig. 4, a typical 10-kg child receives 14 days of continuous intravenous fentanyl at 1.5 mcg kg^−1^ h^−1^, followed by an 8-day period of weaning. Due to the high value of k_dep_ of fentanyl, *C*_plasma_ and *C*_dependence_ closely follow each other; therefore, the risk of IWS is primarily associated with the rate of decrease in *C*_plasma_. This figure illustrates that due to these IWS characteristics, fentanyl is best weaned off in small steps to avoid peak risks of IWS. When fentanyl is weaned off in steps of 20% of the initial rate every 48 h (right column), there is an over twofold increase in the peak risk of IWS. The peak risk of IWS is seen when the infusion rate is decreased, as that is when the rate of decrease in *C*_plasma_ is highest. A lower risk of IWS can be expected when applying smaller, but more frequent (every 12 or 24 h) reductions in the fentanyl infusion rate (Fig. 4, left and middle column). This phenomenon is not seen in simulations with morphine, as this drug has a much lower *k*_dep_, and the simulated risk of IWS in scenarios in which morphine is weaned in steps of 48 h is similar to that in scenarios with weaning in steps of 12 h (Supplemental Material 8).

## DISCUSSION

We currently lack a quantitative understanding of how different weaning strategies can affect the risk of IWS over time in children who required prolonged treatment of (combinations of) opioids and/or sedatives. To allow prediction of the risk of IWS over time, we applied a new mechanism-based PKPD modeling approach in a PICU population of children older than 1 month (median age 22 months), who received continuous infusions with opioids and/or sedatives for 5 days or more. The model adequately fitted the observed NRS_withdrawal_ data in this population and quantified the IWS dynamics of fentanyl, morphine, and ketamine. Additionally, we confirmed that the predictions of the model are in agreement with what clinical knowledge dictates: abrupt discontinuation of a continuous infusion will result in an increased risk of IWS, but this risk can be reduced by slowly weaning off the continuous infusion (Fig. 2) (1, 6).

Additional simulations generated new hypotheses that could guide future clinical research on optimal weaning strategies. For the three drugs included in the IWS model, simulations suggested that a longer weaning period should be used when a higher infusion rate is administered before weaning, to have the same IWS risk as patients with a lower infusion rate (Fig. 3, Supplemental Material 6 and 7). Similarly, these results suggest that patients with a relatively low infusion rate might be weaned relatively fast without a high risk of IWS. While these results may seem intuitive, they contradict with most weaning protocols reported in literature. Most weaning protocols tend to stepwise reduce the infusion rate by a set percentage of the original infusion rate, resulting in the same planned weaning period for all patients (e.g., 10% reduction every 24 h) (5, 6, 36).

Regarding fentanyl, from finding that there is a fast rate of dependence development and disappearance, we hypothesize that small frequent (e.g., every 12 h) reductions of the infusion rate could reduce the IWS risk of fentanyl compared to greater but less frequent (e.g., every 48 h) reductions. Interestingly, weaning from morphine is less dependent on the intervals between weaning steps, as the predicted IWS risk from morphine was similar in scenarios with weaning every 12 or 48 h (Supplemental Material 8). These observations might explain clinical observations that the risk of IWS is higher for patients treated with fentanyl than for patients treated with morphine, as the weaning strategy of fentanyl would be less forgiving to sudden large reductions in infusion rate (3, 37–39). Our results suggest that a “small but frequent” approach to fentanyl weaning might reduce the heightened risk for IWS after fentanyl treatment.

Through the simulations discussed here, the model allowed us to explore weaning scenarios that would be not be possible to prospectively study in real patients due to ethical reasons, e.g., sticking to a strict monotherapy treatment and weaning protocol without allowing for any dose adjustments upon medical need. These simulation scenarios demonstrate the utility of the PKPD modeling approach to explore the interplay between pre-weaning drug treatment, weaning strategy, and the risk of IWS. In this paper, for the sake of simplicity, we only showed simulations in which a single drug is responsible for IWS, but the model can also be used to predict the risk of IWS in scenarios with multiple drugs.

We found a large difference in the estimated *k*_dep_ between fentanyl and morphine, although both drugs target the mu-opioid receptor (40). It has been suggested that some differences in pharmacology between opioids originate from a complex interplay between drug distribution in the body and their differential activation profiles of different mu opioid receptor subtypes, which might also explain the differences in *k*_dep_ we observed here (40, 41). Another possible explanation of the difference between fentanyl and morphine is that morphine-3-glucoronide and morphine-6-glucoronide, which are active metabolites of morphine that were not incorporated in the model, may have contributed to the observed IWS dynamics after morphine treatment (40). This might be especially relevant in patients with impaired renal function as this could result in a long half-life and accumulation of these metabolites.

Among the patients analyzed in the current study, 52% had one or more observations of IWS. The reported incidence of IWS in literature varies widely (5–87%), which likely reflects the heterogeneity of both the patient population and the clinical protocols used for both sedation and weaning in different hospitals. Differences in these protocols may impact the applicability of our findings to other clinical settings. Another limitation of the study is the lack of simulation-based diagnostics, which are considered the gold standard model evaluation techniques, but which could not be created because the complex dosing patterns (with up- and downtitration of multiple drugs depending on NRS_withdrawal_ scores) could not be re-created in a simulation (42).

Although we could quantify the impact of three drugs on IWS in children, we cannot exclude a confounding effect from drugs not included in the model because of a lack of statistical significance. These include drugs that might contribute to IWS (e.g., sedatives like midazolam) or drugs used to manage IWS during weaning (e.g., clonidine, methadone) (5). The effects of these drugs on IWS in this study cohort may have been more modest or more variable in nature than the effects of the drugs included in the model. Additionally, the relatively low occurrence of high NRS_withdrawal_ scores in this study cohort limits the statistical power to identify the effects of additional drugs on IWS. By applying our mechanism-based modeling approach in larger datasets, we might obtain a more comprehensive picture of the relative influences of these drugs on IWS and improve the predictive performance of the model. This also includes drugs, like dexmedetomidine, that were not used in this study, but which may also induce IWS after prolonged use (43).

## CONCLUSIONS

We developed a novel mechanism-based PKPD modeling approach to quantify the IWS dynamics of morphine, fentanyl, and ketamine from a large clinical dataset of critically ill children. The simulations performed with this model suggest that with a higher infusion rate before weaning a longer weaning period would be needed to have the same IWS risk as patients with a lower infusion rate. For fentanyl specifically, weaning with small but frequent reductions in the infusion rate might reduce the heightened risk for IWS after fentanyl treatment. The mechanism-based modeling approach introduced here might well be applied in other datasets to quantify the IWS dynamics of other opioids and sedatives.

## Supplementary Information

ESM 1 (PDF 494 kb)

ESM 2 (PDF 215 kb)

ESM 3 (TXT 9 kb)

ESM 4 (DOCX 14 kb)

ESM 5 (PDF 484 kb)

ESM 6 (PDF 334 kb)

ESM 7 (PDF 338 kb)

ESM 8 (PDF 465 kb)
