# Supplementary material for: Towards Evidence-Based Weaning: a Mechanism-Based Pharmacometric Model to Characterize Iatrogenic Withdrawal Syndrome in Critically Ill Children
Source: AAPS J. 2021 May 17;23(4):71. doi: 10.1208/s12248-021-00586-w (PMC8128736; doi:10.1208/s12248-021-00586-w)
Supplement: Supplementary file 1 — (PDF 494 kb) [file 12248_2021_586_MOESM1_ESM.pdf]

Towards evidence-based weaning: a mechanism-based pharmacometric model to characterize iatrogenic withdrawal syndrome in critically-ill children

Sebastiaan C. Goulooze (1,2), Erwin Ista (3), Monique van Dijk (3,4), Dick Tibboel (3), Elke H.J. Krekels (1), Catherijne A.J. Knibbe (1,5)

(1) Division of Systems Biomedicine and Pharmacology, Leiden Academic Centre for Drug Research, Leiden University, Leiden, The Netherlands (2) LAP&P Consultants BV, Leiden, The Netherlands (3) Pediatric Surgery, Erasmus Medical Center-Sophia Children's Hospital, Rotterdam, The Netherlands (4) Division of Nursing Science, Department of Internal Medicine, Erasmus Medical Center, The Netherlands (5) Department of Clinical Pharmacy, St. Antonius Hospital, Nieuwegein, The Netherlands

Supplemental materials

This document contains detailed information on the methods used to predict the drug concentrations over time for the following eight drugs: morphine, fentanyl, ketamine, methadone, midazolam, lorazepam, propofol, clonidine. The information is listed by drug and includes:

- Schematic representation of the structure of the pharmacokinetic (PK) model
- List of parameters used by the PK model
- Equations used to calculate the PK parameters in each individual based on their bodyweight and/or age.
- Supporting literature references for each PK parameter
- Number of dose records for each drug and administration route that is included in the dataset.

Morphine

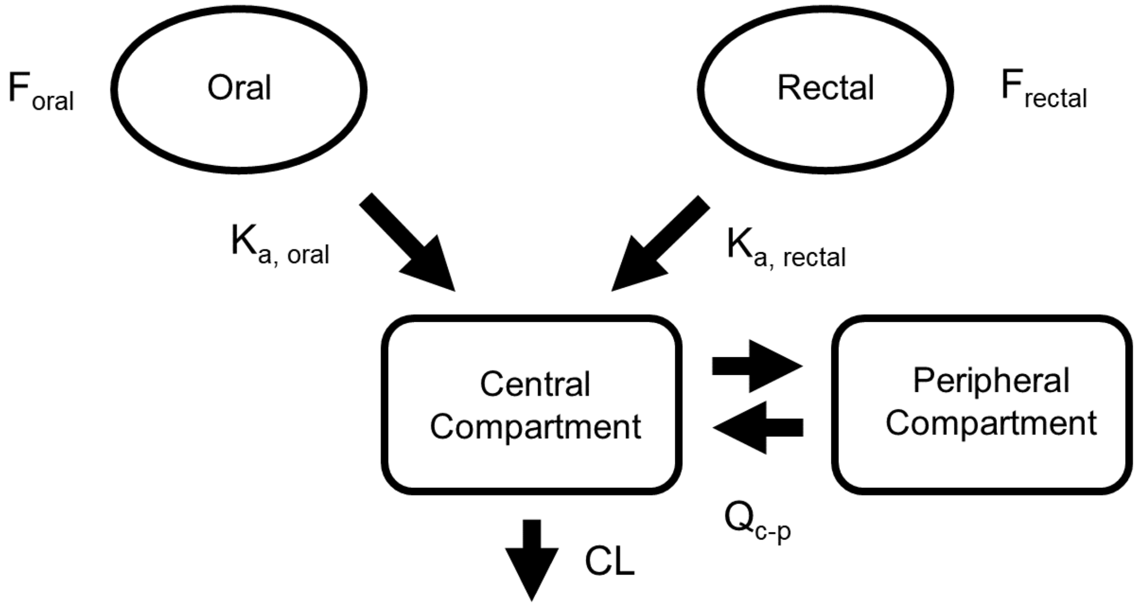

Figure 1. Schematic representation of used pharmacokinetic model to predict the morphine concentrations in plasma over time. Parameters required for this model are: oral bioavailability (F_{oral}), oral absorption rate constant ($K_{a,\ oral}$), rectal bioavailability (F_{rectal}), rectal absorption rate constant ($K_{a,rectal}$), volume of central compartment (V_c), volume of peripheral compartment (V_p), intercompartmental clearance ($Q_{c\text{-}p}$) and drug clearance (CL). One intrathecal dose of morphine was incorporated as the equipotent intravenous (i.e. 100-fold higher amount) dose in the central compartment.

Table 1. Parameter values used to predict morphine concentrations and supporting publications

Parameter (unit)	Value	Ref.	Ref. type
F_{oral} (-)	0.34	(1, 2)	Adult PK
$K_{a,oral}$ (h^{-1})	6.0	(1, 2)	Adult PK
F_{rectal} (-)	0.533	(3, 4)	Adult PK
$K_{a,rectal}$ (h^{-1})	1.0	(4)	Adult PK
V_c (L)	$29.3 \times \left(\frac{BW}{70}\right)$	(5)	Pediatric popPK
V_p (L)	$155 \times \left(\frac{BW}{70}\right)$	(5)	Pediatric popPK
$Q_{c\text{-}p}$ (L/h)	$252 \times \left(\frac{BW}{70}\right)$	(5)	Pediatric popPK
CL(L/h)	$3.6 \times \left(\frac{BW}{70}\right) + 100.2 \times \left(\frac{BW}{70}\right)^{\left(1.56 - 0.67 \times \frac{BW^{3.61}}{3.89^{3.61} + BW^{3.61}}\right)}$	(5)	Pediatric popPK

BW = patient body weight in kilograms

Additional notes:

The dataset for this analysis contained a total of 6336 morphine dosing records of which 6327 (99.9%) were intravenous doses, 5 (0.08%) were oral doses, 3 (0.05%) were rectal doses, and 1 was an intrathecal dose (0.02%).

Fentanyl

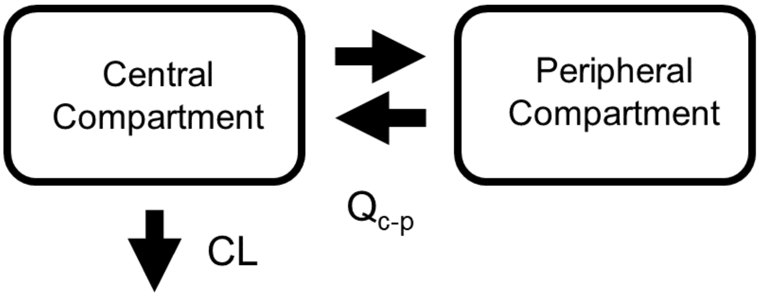

Figure 2. Schematic representation of used pharmacokinetic model to predict the fentanyl concentrations in plasma over time. Parameters required for this model are: volume of central compartment (V_c), volume of peripheral compartment (V_p), intercompartmental clearance (Q_{c-p}) and drug clearance (CL).

Table 1. Parameter values used to predict fentanyl concentrations and supporting publications

Parameter (unit)	Value	Ref.	Ref. type
V_c (L)	$if\ age < 5\ years; 529.1 \times \frac{BW}{70}$<br>$if\ age \geq 5\ years;\ 203 \times \frac{BW}{92}$	(6)<br>(7, 8)	Pediatric popPK<br>Adult popPK (allometrically scaled)
V_p (L)	$if\ age < 5\ years;\ 227.3 \times \frac{BW}{70}$<br>$if\ age \geq 5\ years;\ 523 \times \frac{BW}{92}$	(6)<br>(7, 8)	Pediatric popPK<br>Adult popPK (allometrically scaled)
Q_{c-p} (L/h)	$if\ age < 5\ years; 7.8 \times \left(\frac{BW}{70}\right)^{0.75}$<br>$if\ age \geq 5\ years;\ 55 \times \left(\frac{BW}{92}\right)^{0.75}$	(6)<br>(7, 8)	Pediatric popPK<br>Adult popPK (allometrically scaled)
CL (L/h)	$if\ age < 5\ years;\ 39.6 \times \left(\frac{BW}{70}\right)^{0.75}$<br>$if\ age \geq 5\ years;\ 35 \times \left(\frac{BW}{92}\right)^{0.75}$	(6)<br>(7, 8)	Pediatric popPK<br>Adult popPK (allometrically scaled)

BW = patient body weight in kilograms

Additional notes:

All 1811 fentanyl dosing records in the dataset concerned intravenous administrations. The PK parameter estimates from Choi (7) were allometric scaled (centering on 92 kg, the median bodyweight of the adult patient population) to improve the extrapolation potential of the model.

Ketamine

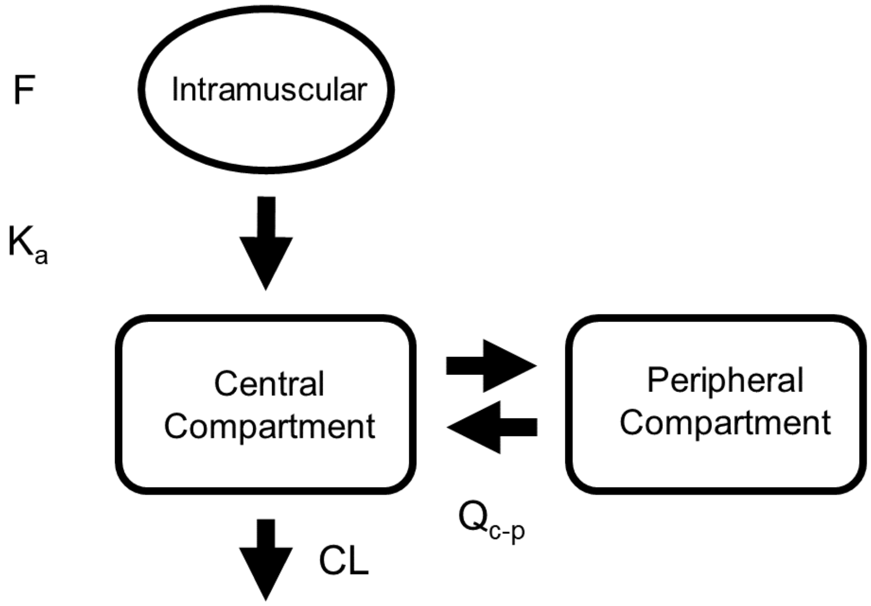

Figure 3. Schematic representation of used pharmacokinetic model to predict the ketamine concentrations in plasma over time. Parameters required for this model are: intramuscular bioavailability (F), intramuscular absorption rate constant (K_a), volume of central compartment (V_c), volume of peripheral compartment (V_p), intercompartmental clearance (Q_{c-p}) and drug clearance (CL).

Table 1. Parameter values used to predict ketamine concentrations and supporting publications

Parameter (unit)	Value	Ref.	Ref. type
F (-)	1.0	(9)	Pediatric popPK
K_a (h^{-1})	1.98	(9)	Pediatric popPK
V_c (L)	$57.3\times\left(\frac{BW}{70}\right)$	(10)	Pediatric popPK
V_p (L)	$152\times\left(\frac{BW}{70}\right)$	(10)	Pediatric popPK
Q_{c-p} (L/h)	$73.2\times\left(\frac{BW}{70}\right)^{0.75}$	(10)	Pediatric popPK
CL (L/h)	$60.6\times\left(\frac{BW}{70}\right)^{0.75}$	(10)	Pediatric popPK

BW = patient body weight in kilograms

Additional notes:

The data analysis contained a total of 2875 ketamine dosing records, of which 2874 (>99.9%) where intravenous doses and a single intramuscular ketamine dose.

Methadone

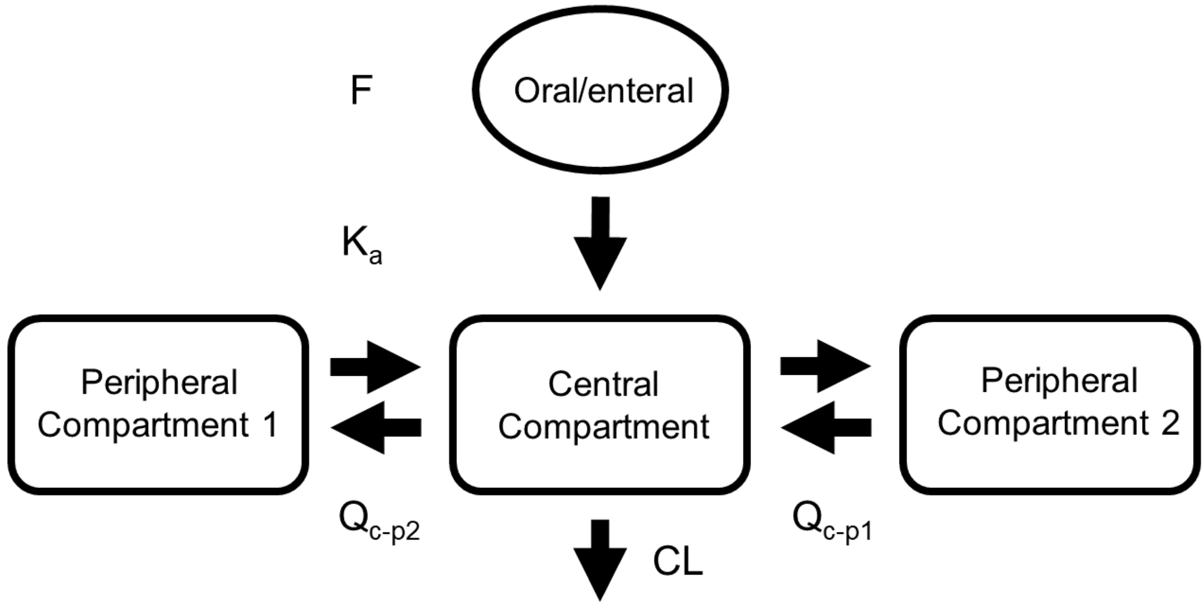

Figure 4. Schematic representation of used pharmacokinetic model to predict the methadone concentrations in plasma over time. Parameters required for this model are: oral/enteral bioavailability (F), oral/enteral absorption rate constant (K_a), volume of central compartment (V_c), volume of the two peripheral compartments (V_{p1} and V_{p2}), intercompartmental clearances to the peripheral compartments ($Q_{c\text{-}p1}$ and $Q_{c\text{-}p2}$) and drug clearance (CL).

Table 1. Parameter values used to predict methadone concentrations and supporting publications

Parameter (unit)	Value	Ref.	Ref. type
F (-)	0.86	(11)	Adult PK
K_a (h^{-1})	=1.0	(11)	Adult PK
V_c (L)	$21.5 \times \left(\frac{BW}{70}\right) \times \left(1 + 1.63 \times e^{\left(-1 \times \left(\frac{AGE}{7}+3\right) \times \frac{\ln(2)}{107}\right)}\right)$	(12)	Pediatric popPK
V_{p1} (L)	$75.1 \times \left(\frac{BW}{70}\right)$	(12)	Pediatric popPK
$Q_{c\text{-}p1}$ (L/h)	$324.6 \times \left(\frac{BW}{70}\right)^{0.75}$	(12)	Pediatric popPK
V_{p2} (L)	$484 \times \left(\frac{BW}{70}\right)$	(12)	Pediatric popPK
$Q_{c\text{-}p2}$ (L/h)	$136.4 \times \left(\frac{BW}{70}\right)^{0.75}$	(12)	Pediatric popPK
CL (L/h)	$9.45 \times \left(\frac{BW}{70}\right)^{0.75}$	(12)	Pediatric popPK

BW = patient body weight in kilograms, AGE = patient postnatal age in days

Additional notes:

All 490 methadone dosing records in the dataset concern oral/enteral administrations. The effect of age on clearance in Ward *et al*. (12) is parameterized as a function of post-menstrual age, rather than postnatal age. However, since the pregnancy duration was generally not recorded for older children in the present dataset, the original formula was re-parameterized to postnatal age.

Midazolam

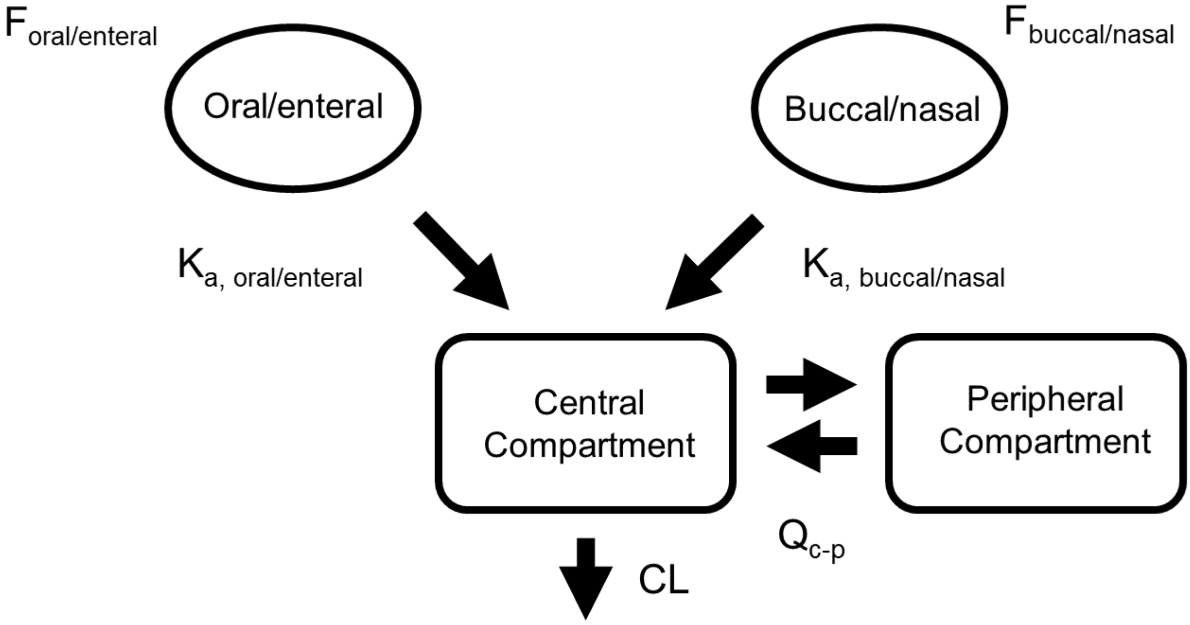

Figure 5. Schematic representation of used pharmacokinetic model to predict the midazolam concentrations in plasma over time. Parameters required for this model are: oral/enteral bioavailability ($F_{oral/enteral}$), oral/enteral absorption rate constant ($K_{a,oral/enteral}$), buccal/nasal bioavailability ($F_{buccal/nasal}$), buccal/nasal absorption rate constant ($K_{a,buccal/nasal}$), volume of central compartment (V_c), volume of peripheral compartment (V_p), intercompartmental clearance (Q_{c-p}) and drug clearance (CL).

Table 1. Parameter values used to predict midazolam concentrations and supporting publications

Parameter (unit)	Value	Ref.	Ref. type
$F_{oral/enteral}$ (-)	0.27	(13)	Pediatric PK
$K_{a,oral/enteral}$ (h^{-1})	1	(13)	Pediatric PK
$F_{buccal/nasal}$ (-)	0.8	(14, 15)	Adult PK
$K_{a,buccal/nasal}$ (h^{-1})	3	(14, 15)	Adult PK
V_c (L)	$3.58 \times \left(\frac{BW}{5}\right)^{1.32}$	(16)	Pediatric popPK
V_p (L)	5.35	(16)	Pediatric popPK
Q_{c-p} (L/h)	1.57	(16)	Pediatric popPK
CL (L/h)	$1.11 \times \left(\frac{BW}{5}\right)^{0.828}$	(16)	Pediatric popPK

BW = patient body weight in kilograms

Additional notes:

The data analysis contained a total of 11559 midazolam dosing records, of which 10853 (93.9%) where intravenous doses, 675 (5.8%) oral/enteral doses and 31 (0.3%) buccal/nasal doses. The base model reported by Vet *et al.* (16) was used, as the covariates included in the final model (CRP concentrations and number of organs failing) were not available in the current dataset.(16)

Lorazepam

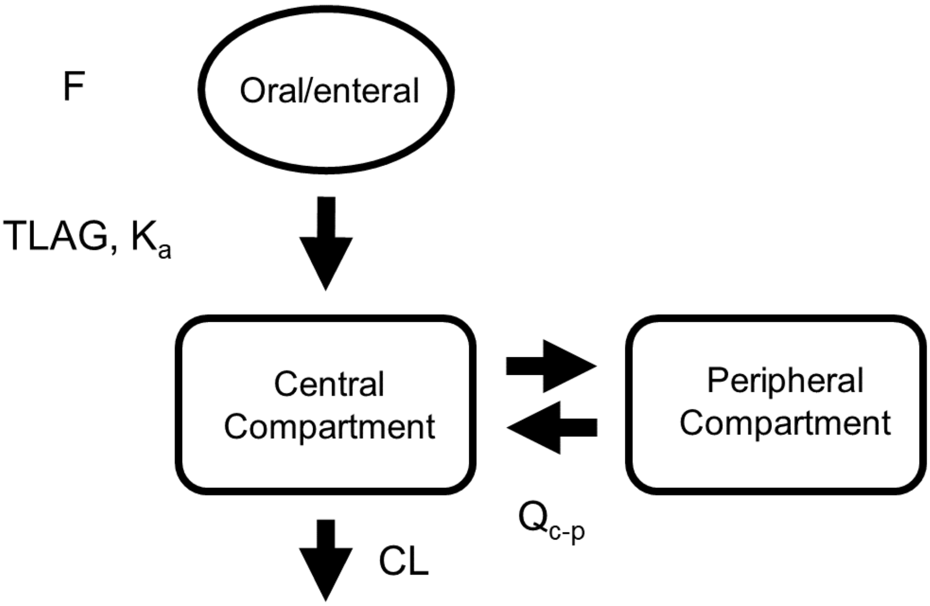

Figure 6. Schematic representation of used pharmacokinetic model to predict the lorazepam concentrations in plasma over time. Parameters required for this model are: oral/enteral bioavailability (F), oral/enteral absorption rate constant (K_a), oral/enteral absorption lag time (TLAG), volume of central compartment (V_c), volume of peripheral compartment (V_p), intercompartmental clearance (Q_{c-p}) and drug clearance (CL).

Table 1. Parameter values used to predict morphine concentrations and supporting publications

Parameter (unit)	Value	Ref.	Ref. type
F (-)	0.93	(17)	Adult PK
K_a (h^{-1})	1.04	(17)	Adult PK
TLAG (h)	0.27	(17)	Adult PK
V_c (L)	$0.879 \times BW$	(18)	Pediatric popPK
V_p (L)	$0.476 \times BW$	(18)	Pediatric popPK
Q_{c-p} (L/h)	$1.45 \times \left(\frac{BW}{70}\right)^{0.75}$	(18)	Pediatric popPK
CL (L/h)	$0.115 \times \left(\frac{BW}{70}\right)^{0.75} \times \left(\frac{AGE}{1716}\right)^{0.133}$	(18)	Pediatric popPK

BW = patient body weight in kilograms, AGE = patient postnatal age in days

Additional notes:

The dataset contained a total of 1068 lorazepam dosing records, which included 430 intravenous administrations (40.3%) and 638 (59.7%) oral/enteral administrations.

Propofol

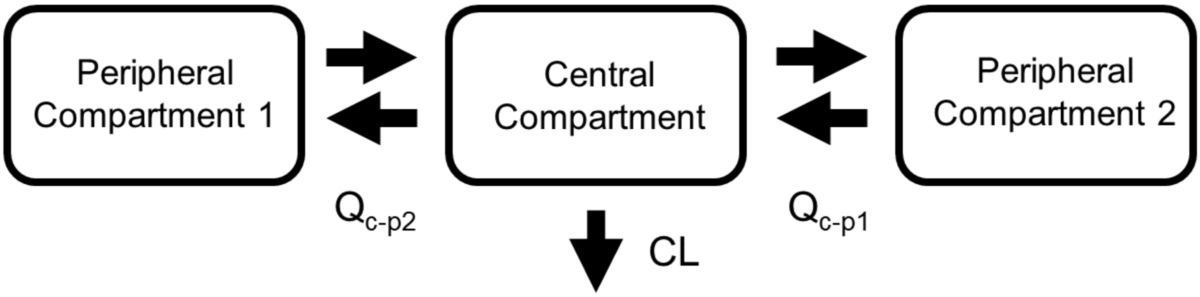

Figure 7. Schematic representation of used pharmacokinetic model to predict the propofol concentrations in plasma over time. Parameters required for this model are: volume of central compartment (V_c), volume of the two peripheral compartments (V_{p1} and V_{p2}), intercompartmental clearances to the peripheral compartments ($Q_{c\text{-}p1}$ and $Q_{c\text{-}p2}$) and drug clearance (CL).

Table 1. Parameter values used to predict propofol concentrations and supporting publications

Parameter (unit)	Value	Ref.	Ref. type
V_c (L)	$0.584 \times BW$	(19)	Pediatric popPK
V_{p1} (L)	$1.36 \times BW$	(19)	Pediatric popPK
$Q_{c\text{-}p1}$ (L/h)	$0.96 \times BW$	(19)	Pediatric popPK
V_{p2} (L)	$103 + 5.67 \times BW$	(19)	Pediatric popPK
$Q_{c\text{-}p2}$ (L/h)	$0.798 \times BW$	(19)	Pediatric popPK
CL (L/h)	$1.81 \times BW$	(19)	Pediatric popPK

Additional notes:

All 989 dosing records for propofol in the dataset concerned intravenous administrations.

Clonidine

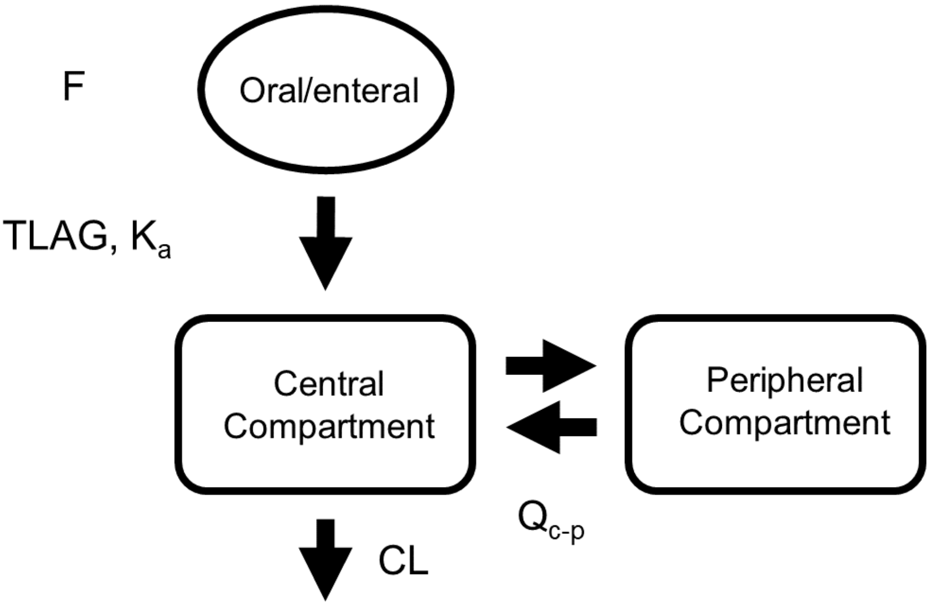

Figure 8. Schematic representation of used pharmacokinetic model to predict the clonidine concentrations in plasma over time. Parameters required for this model are: oral/enteral bioavailability (F), oral/enteral absorption rate constant (K_a), oral/enteral absorption lag time (TLAG),volume of central compartment (V_c), volume of peripheral compartment (V_p), intercompartmental clearance (Q_{c-p}) and drug clearance (CL).

Table 1. Parameter values used to predict clonidine concentrations and supporting publications

Parameter (unit)	Value	Ref.	Ref. type
F (-)	0.554	(20)	Pediatric popPK
K_a (h^{-1})	1.53	(20)	Pediatric popPK
TLAG (h)	0.148	(20)	Pediatric popPK
V_c (L)	$62.5 \times \left(\frac{BW}{70}\right)$	(21)	Pediatric popPK
V_p (L)	$119 \times \left(\frac{BW}{70}\right)$	(21)	Pediatric popPK
Q_{c-p} (L/h)	$157 \times \left(\frac{BW}{70}\right)^{0.75}$	(21)	Pediatric popPK
CL (L/h)	$14.6 \times \left(\frac{BW}{70}\right)^{0.75} \times (1 - 0.738 \times e^{\left(-1\times\left(\frac{AGE}{7}\right)\times\frac{\ln(2)}{25.7}\right)}$	(21)	Pediatric popPK

Additional notes

The dataset contained a total of 4050 clonidine dosing records, which included 3247 (80.2%) intravenous dosing records, and 803 (19.8%) oral/enteral dosing records.

References

(1) Hunt, A., Joel, S., Dick, G. & Goldman, A. Population pharmacokinetics of oral morphine and its glucuronides in children receiving morphine as immediate-release liquid or sustained-release tablets for cancer pain. *J Pediatr* **135**, 47-55 (1999).

(2) Poulain, P. *et al.* Relative bioavailability of controlled release morphine tablets (MST continus) in cancer patients. *Br J Anaesth* **61**, 569-74 (1988).

(3) Jonsson, T., Christensen, C.B., Jordening, H. & Frolund, C. The Bioavailability of Rectally Administered Morphine. *Pharmacol Toxicol* **62**, 203-5 (1988).

(4) Moolenaar, F., Visser, J., Leuverman, A. & Schoonen, B.J.M. Bioavailability of Morphine from Suppositories. *Int J Pharm* **45**, 161-4 (1988).

(5) Wang, C. *et al.* Developmental changes in morphine clearance across the entire paediatric age range are best described by a bodyweight-dependent exponent model. *Clin Drug Investig* **33**, 523-34 (2013).

(6) Van Driest, S.L. *et al.* Pragmatic pharmacology: population pharmacokinetic analysis of fentanyl using remnant samples from children after cardiac surgery. *Br J Clin Pharmacol* **81**, 1165-74 (2016).

(7) Choi, L. *et al.* Population Pharmacokinetics of Fentanyl in the Critically Ill. *Crit Care Med* **44**, 64-72 (2016).

(8) Calvier, E.A. *et al.* Allometric Scaling of Clearance in Paediatric Patients: When Does the Magic of 0.75 Fade? *Clin Pharmacokinet* **56**, 273-85 (2017).

(9) Brunette, K.E., Anderson, B.J., Thomas, J., Wiesner, L., Herd, D.W. & Schulein, S. Exploring the pharmacokinetics of oral ketamine in children undergoing burns procedures. *Paediatr Anaesth* **21**, 653-62 (2011).

(10) Elkomy, M.H., Drover, D.R., Hammer, G.B., Galinkin, J.L. & Ramamoorthy, C. Population pharmacokinetics of ketamine in children with heart disease. *Int J Pharm* **478**, 223-31 (2015).

(11) Dale, O., Sheffels, P. & Kharasch, E.D. Bioavailabilities of rectal and oral methadone in healthy subjects. *Br J Clin Pharmacol* **58**, 156-62 (2004).

(12) Ward, R.M. *et al.* The pharmacokinetics of methadone and its metabolites in neonates, infants, and children. *Paediatr Anaesth* **24**, 591-601 (2014).

(13) Payne, K., Mattheyse, F.J., Liebenberg, D. & Dawes, T. The pharmacokinetics of midazolam in paediatric patients. *Eur J Clin Pharmacol* **37**, 267-72 (1989).

(14) Schwagmeier, R., Alincic, S. & Striebel, H.W. Midazolam pharmacokinetics following intravenous and buccal administration. *Br J Clin Pharmacol* **46**, 203-6 (1998).

(15) Bjorkman, S., Rigemar, G. & Idvall, J. Pharmacokinetics of midazolam given as an intranasal spray to adult surgical patients. *Br J Anaesth* **79**, 575-80 (1997).

(16) Vet, N.J. *et al.* Inflammation and Organ Failure Severely Affect Midazolam Clearance in Critically Ill Children. *Am J Respir Crit Care Med* **194**, 58-66 (2016).

(17) Greenblatt, D.J. *et al.* Pharmacokinetics and bioavailability of intravenous, intramuscular, and oral lorazepam in humans. *J Pharm Sci* **68**, 57-63 (1979).

(18) Gonzalez, D. *et al.* Population Pharmacokinetics and Exploratory Pharmacodynamics of Lorazepam in Pediatric Status Epilepticus. *Clin Pharmacokinet* **56**, 941-51 (2017).

(19) Rigby-Jones, A.E., Nolan, J.A., Priston, M.J., Wright, P.M., Sneyd, J.R. & Wolf, A.R. Pharmacokinetics of propofol infusions in critically ill neonates, infants, and children in an intensive care unit. *Anesthesiology* **97**, 1393-400 (2002).

(20) Larsson, P. *et al.* Oral bioavailability of clonidine in children. *Paediatr Anaesth* **21**, 335-40 (2011).

(21) Potts, A.L., Larsson, P., Eksborg, S., Warman, G., Lonnqvist, P.A. & Anderson, B.J. Clonidine disposition in children; a population analysis. *Paediatr Anaesth* **17**, 924-33 (2007).
