# Supplementary material for: Towards Evidence-Based Weaning: a Mechanism-Based Pharmacometric Model to Characterize Iatrogenic Withdrawal Syndrome in Critically Ill Children
Source: AAPS J. 2021 May 17;23(4):71. doi: 10.1208/s12248-021-00586-w (PMC8128736; doi:10.1208/s12248-021-00586-w)
Supplement: Supplementary file 2 — (PDF 215 kb) [file 12248_2021_586_MOESM2_ESM.pdf]

Towards evidence-based weaning: a mechanism-based pharmacometric model to characterize iatrogenic withdrawal syndrome in critically-ill children

Sebastiaan C. Goulooze (1,2), Erwin Ista (3), Monique van Dijk (3,4), Dick Tibboel (3), Elke H.J. Krekels (1), Catherijne A.J. Knibbe (1,5)

(1) Division of Systems Biomedicine and Pharmacology, Leiden Academic Centre for Drug Research, Leiden University, Leiden, The Netherlands (2) LAP&P Consultants BV, Leiden, The Netherlands (3) Pediatric Surgery, Erasmus Medical Center-Sophia Children's Hospital, Rotterdam, The Netherlands (4) Division of Nursing Science, Department of Internal Medicine, Erasmus Medical Center, The Netherlands (5) Department of Clinical Pharmacy, St. Antonius Hospital, Nieuwegein, The Netherlands

Supplemental materials

This document provides additional information on the equations that were used in the mechanism-based IWS model to incorporate the effect of drugs (e.g. clonidine) that might reduce the iatrogenic withdrawal that originates from another drug. Also provided are the equations that were used to explore an interaction between withdrawal from drugs with the same mechanism of action into the model (e.g. morphine and fentanyl). Finally, a model is also provided that adds an additional tolerance compartment (in addition to the $C_{dependence}$ compartment described with Equation 1). In all cases, the equation given in this document replaced Equation 2 in the main manuscript.

Linear model for drug lowering the effect on IWS severity by another drug

$$Effect_{fentanyl} = Slope_{fentanyl} * \left(C_{dependence,fentanyl} - C_{plasma,fentanyl}\right) - C_{plasma,clonidine} * Slope_{clonidine}$$

Where $Effect_{fentanyl}$ was fixed to zero if it this equation resulted in a negative value for $Effect_{fentanyl}$. This model was also used to test whether morphine would lower fentanyl-associated withdrawal and vice versa.

E_{max} model for drug lowering the effect on IWS severity by another drug

$$Effect_{fentanyl} = Slope_{fentanyl} * \left(C_{dependence,fentanyl} - C_{plasma,fentanyl}\right) * \left(1 - \frac{Emax * C_{plasma,clonidine}}{C_{plasma,clonidine} + EC50}\right)$$

Where E_{max} is the maximum effect of clonidine on fentanyl withdrawal, and $EC50$ is the clonidine concentration at which the effect reaches 50% of the E_{max}. $Effect_{fentanyl}$ was fixed to zero if it this equation resulted in a negative value for $Effect_{fentanyl}$.

Combined model for effects of two drugs from the same class on IWS severity

$$Effect_{opioids} = Slope_{fentanyl} * \left(C_{dependence,fentanyl} - C_{plasma,fentanyl}\right) + Slope_{morphine} * \left(C_{dependence,morphine} - C_{plasma,morphine}\right)$$

Where $Effect_{opioids}$ was fixed to zero if it this equation resulted in a negative value of $Effect_{opioids}$. The difference between this model and the additive model used in the final PKPD model is that this model allows a 'surplus' of fentanyl ($C_{plasma} > C_{dependence}$) to compensate for a 'lack' of morphine ($C_{plasma} < C_{dependence}$), and vice versa. In the additive model, the effect of different drugs are calculated separately according to equation 2, and fixed to zero if $C_{dependence}$ is not higher than C_{plasma}.

Combined dependence and tolerance model for a single drug

In this model, the development of dependence and tolerance are modelled separately. An additional (hypothetical) compartment is added, which tracks the development of tolerance (TOLER) over time. The differential equation defining this compartment is:

$$\frac{dTOLER}{dt} = k_{TOL} * (C_{plasma} - TOLER)$$

Where k_{TOL} is the estimated rate of tolerance development and C_{plasma} is the drug concentration in plasma. As TOLER increases, the effective plasma concentration is reduced:

$$C_{plasma,eff} = C_{plasma} * \left(1 - \frac{TOLER}{TOLER + IC50}\right)$$

Where IC50 is the parameter that controls the impact of TOLER on $C_{plasma,eff}$. In the model with the tolerance compartment, the effect on IWS severity is calculated using $C_{plasma,eff}$ rather than C_{plasma}:

$$Effect_j = Slope_j * (C_{dependence} - C_{plasma,eff})$$

Similar to the other equations in the Supplemental Material, $Effect_j$ was fixed to zero if it this equation resulted in a negative value for $Effect_j$.

The model described in this section adds two additional estimated parameters to the IWS model, k_{TOL} and IC50. An important feature of the model described here is that at steady state (when $C_{dependence} = C_{plasma}$) the effect of TOLER will result in a nonzero effect on IWS severity. This is different from the simpler IWS model described in the main text (i.e. the model without a tolerance compartment), for which $Effect_j$ is always zero at steady state (when $C_{dependence} = C_{plasma}$).
