# Supplementary material for: Towards Evidence-Based Weaning: a Mechanism-Based Pharmacometric Model to Characterize Iatrogenic Withdrawal Syndrome in Critically Ill Children
Source: AAPS J. 2021 May 17;23(4):71. doi: 10.1208/s12248-021-00586-w (PMC8128736; doi:10.1208/s12248-021-00586-w)
Supplement: Supplementary file 4 — (DOCX 14 kb) [file 12248_2021_586_MOESM4_ESM.docx]

Table S1. Overview of key steps during model development from base to final model.

| Key model # | Description | # parameters | OFV |
| --- | --- | --- | --- |
| 1 | Base model. Truncated Poisson model with estimated overdispersion parameter and IIV on baseline IWS severity. No effects of drugs on IWS. | 3 | 4874.144 |
| 2 | As model 1, but estimates Markovian probability inflation parameters π0\|0 and π0\|x | 5 | 4528.465 |
| 3 | As model 2, but also estimates Markovian probability inflation parameters π±1 | 6 | 4522.587 |
| 4 | As model 3, but also estimates Markovian probability inflation parameters π±2 | 7 | 4493.626 |
| 5 | As model 4, but sets π±1 = π±2. | 6 | 4493.738 |
| 6 | As model 5, but adds estimated effect of fentanyl on IWS | 8 | 4461.315 |
| 7 | As model 6, but adds estimated effect of morphine on IWS | 10 | 4454.880 |
| 8 | As model 7, but adds estimated effect of ketamine on IWS | 12 | 4444.475 |

OFV =objective function value; IIV = inter-individual variability; IWS = iatrogenic withdrawal syndrome. π0|0 = probability inflation of observing the same score as before if the previous score was zero, π0|x = probability inflation of observing the same score as before if the previous score was not zero, π±1 = probability inflation of observing a score that is 1 point higher or lower than the previous score, π±2 = probability inflation of observing a score that is 2 points higher or lower than the previous score.
