# Supplementary material for: Towards Evidence-Based Weaning: a Mechanism-Based Pharmacometric Model to Characterize Iatrogenic Withdrawal Syndrome in Critically Ill Children
Source: AAPS J. 2021 May 17;23(4):71. doi: 10.1208/s12248-021-00586-w (PMC8128736; doi:10.1208/s12248-021-00586-w)
Supplement: Supplementary file 5 — (PDF 484 kb) [file 12248_2021_586_MOESM5_ESM.pdf]

Towards evidence-based weaning: a mechanism-based pharmacometric model to characterize iatrogenic withdrawal syndrome in critically-ill children

Sebastiaan C. Goulooze (1,2), Erwin Ista (3), Monique van Dijk (3,4), Dick Tibboel (3), Elke H.J. Krekels (1), Catherijne A.J. Knibbe (1,5)

(1) Division of Systems Biomedicine and Pharmacology, Leiden Academic Centre for Drug Research, Leiden University, Leiden, The Netherlands (2) LAP&P Consultants BV, Leiden, The Netherlands (3) Pediatric Surgery, Erasmus Medical Center-Sophia Children's Hospital, Rotterdam, The Netherlands (4) Division of Nursing Science, Department of Internal Medicine, Erasmus Medical Center, The Netherlands (5) Department of Clinical Pharmacy, St. Antonius Hospital, Nieuwegein, The Netherlands

Supplemental Material 5: Supplemental Figure S1

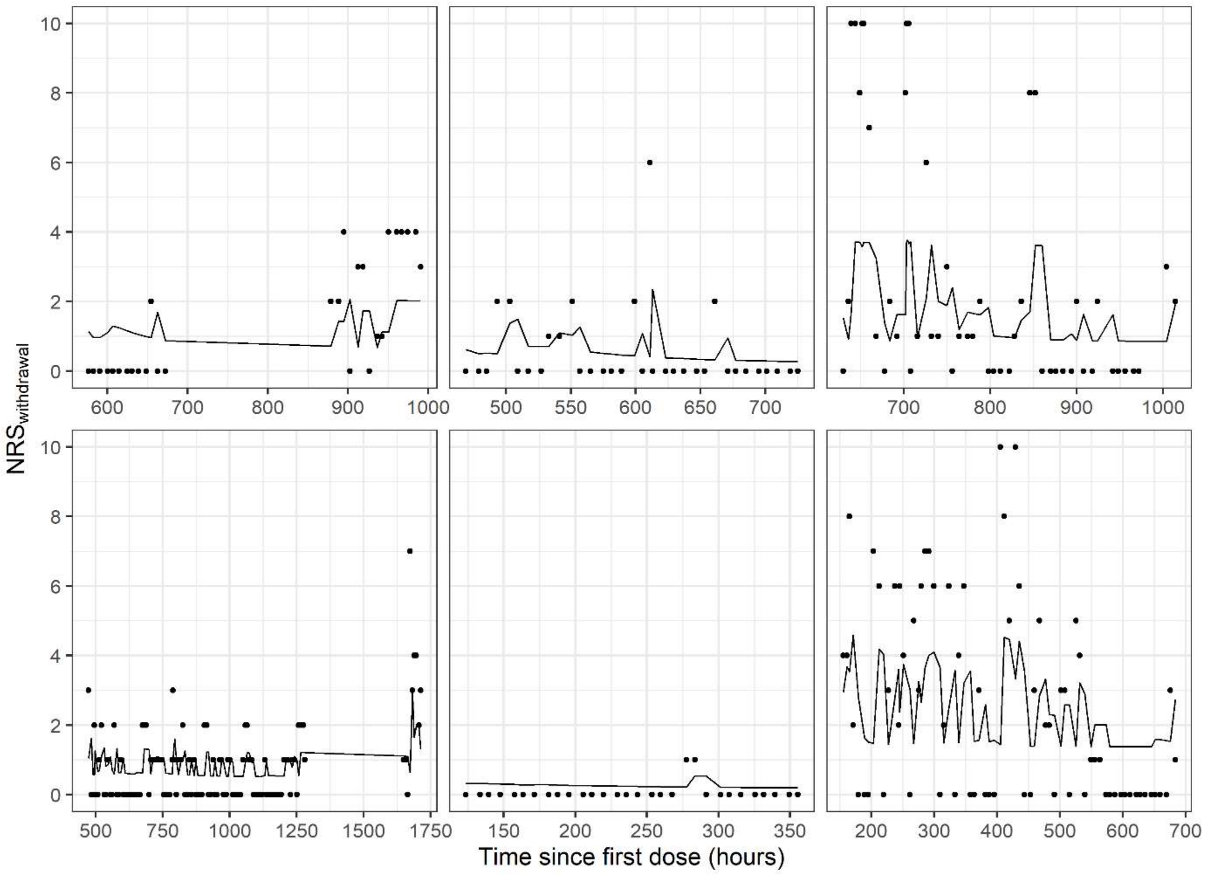

Figure S1. Individual fit plots of six randomly selected subjects with more than 25 observations during the study. The dots indicate the observed $NRS_{withdrawal}$ scores, while the solid line indicates the expected $NRS_{withdrawal}$ score, which is based on (1) the individual predicted baseline iatrogenic withdrawal syndrome (IWS) risk (2) the predicted drug effect on IWS (3) the Markovian impact of the previous observation.
