# Supplementary material for: Towards Evidence-Based Weaning: a Mechanism-Based Pharmacometric Model to Characterize Iatrogenic Withdrawal Syndrome in Critically Ill Children
Source: AAPS J. 2021 May 17;23(4):71. doi: 10.1208/s12248-021-00586-w (PMC8128736; doi:10.1208/s12248-021-00586-w)
Supplement: Supplementary file 6 — (PDF 334 kb) [file 12248_2021_586_MOESM6_ESM.pdf]

Towards evidence-based weaning: a mechanism-based pharmacometric model to characterize iatrogenic withdrawal syndrome in critically-ill children

Sebastiaan C. Goulooze (1,2), Erwin Ista (3), Monique van Dijk (3,4), Dick Tibboel (3), Elke H.J. Krekels (1), Catherijne A.J. Knibbe (1,5)

(1) Division of Systems Biomedicine and Pharmacology, Leiden Academic Centre for Drug Research, Leiden University, Leiden, The Netherlands (2) LAP&P Consultants BV, Leiden, The Netherlands (3) Pediatric Surgery, Erasmus Medical Center-Sophia Children's Hospital, Rotterdam, The Netherlands (4) Division of Nursing Science, Department of Internal Medicine, Erasmus Medical Center, The Netherlands (5) Department of Clinical Pharmacy, St. Antonius Hospital, Nieuwegein, The Netherlands

Supplemental Material 6: Figure S2

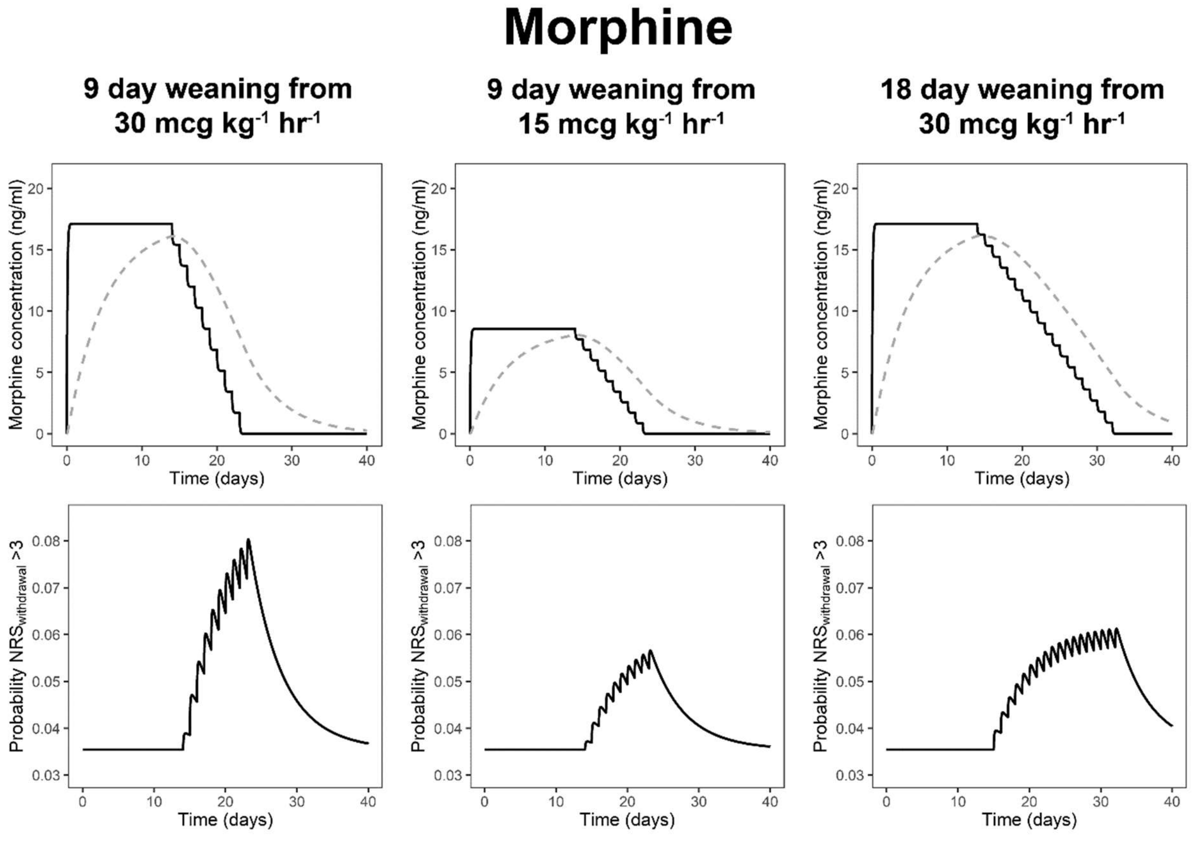

Figure S2. The impact of the morphine infusion rate (30 or 15 mcg kg^{-1} hr^{-1}) during a 14-day treatment period and weaning duration (9 or 18 day weaning) on the risk of iatrogenic withdrawal syndrome (IWS) during weaning in a typical patient with a 10 kg body weight. The top row shows the simulated morphine concentrations in plasma (C_{plasma}, solid black line) and morphine concentrations that the child has become dependent on ($C_{dependence}$, dashed grey line). The bottom row shows the predicted probability of an $NRS_{withdrawal}$ score above 3, which indicates IWS. In all scenarios simulated here, the time between consecutive weaning steps is 24 hours.
