# Supplementary material for: Towards Evidence-Based Weaning: a Mechanism-Based Pharmacometric Model to Characterize Iatrogenic Withdrawal Syndrome in Critically Ill Children
Source: AAPS J. 2021 May 17;23(4):71. doi: 10.1208/s12248-021-00586-w (PMC8128736; doi:10.1208/s12248-021-00586-w)
Supplement: Supplementary file 7 — (PDF 338 kb) [file 12248_2021_586_MOESM7_ESM.pdf]

Towards evidence-based weaning: a mechanism-based pharmacometric model to characterize iatrogenic withdrawal syndrome in critically-ill children

Sebastiaan C. Goulooze (1,2), Erwin Ista (3), Monique van Dijk (3,4), Dick Tibboel (3), Elke H.J. Krekels (1), Catherijne A.J. Knibbe (1,5)

(1) Division of Systems Biomedicine and Pharmacology, Leiden Academic Centre for Drug Research, Leiden University, Leiden, The Netherlands (2) LAP&P Consultants BV, Leiden, The Netherlands (3) Pediatric Surgery, Erasmus Medical Center-Sophia Children’s Hospital, Rotterdam, The Netherlands (4) Division of Nursing Science, Department of Internal Medicine, Erasmus Medical Center, The Netherlands (5) Department of Clinical Pharmacy, St. Antonius Hospital, Nieuwegein, The Netherlands

Supplemental Material 7: Supplemental Figure S3

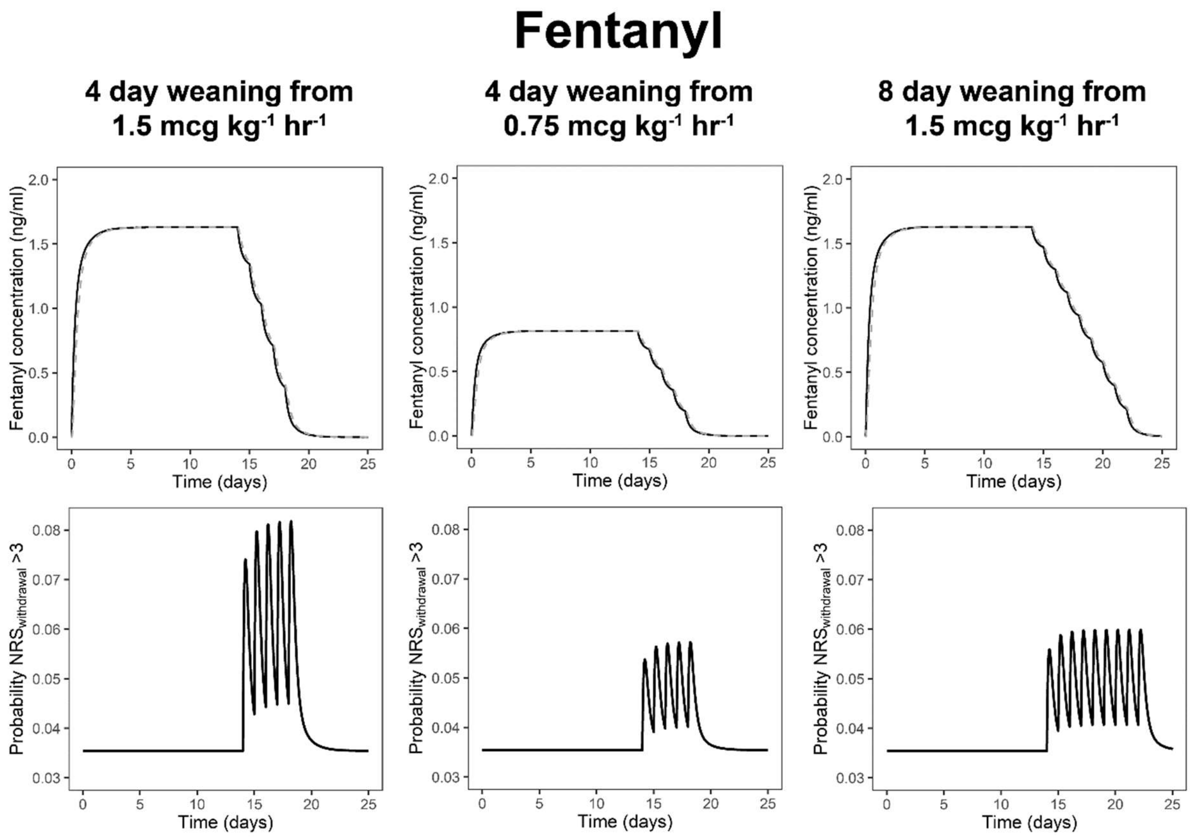

Figure S3. The impact of the fentanyl infusion rate (1.5 or 0.75 mcg kg^{-1} hr^{-1}) during a 14-day treatment period and weaning duration (4 or 8 day weaning) on the risk of iatrogenic withdrawal syndrome (IWS) during weaning in a typical patient with a 10 kg body weight. The top row shows the simulated fentanyl concentrations in plasma (C_{plasma}, solid black line) and fentanyl concentrations that the child has become dependent on ($C_{dependence}$, dashed grey line). Due to the high dependence rate of fentanyl (k_{dep} = 0.265 h^{-1}), C_{plasma} and $C_{dependence}$ closely follow each other. The bottom row shows the predicted probability of an $NRS_{withdrawal}$ score above 3, which indicates IWS. In all scenarios simulated here, the time between consecutive weaning steps is 24 hours.
